# Genomic analysis of demographic history and ecological niche modeling in the endangered Chinese Grouse *Tetrastes sewerzowi*

**DOI:** 10.1186/s12864-020-06957-5

**Published:** 2020-08-27

**Authors:** Kai Song, Bin Gao, Peter Halvarsson, Yun Fang, Ying-Xin Jiang, Yue-Hua Sun, Jacob Höglund

**Affiliations:** 1 Animal Ecology, Department of Ecology and Genetics, Evolutionary Biology Centre, Uppsala University, Norbyvägen 18D, 75236 Uppsala, Sweden; 2 Key Laboratory of Animal Ecology and Conservation Biology, Institute of Zoology, Chinese Academy of Sciences, Beijing, 100101 P. R. China; 3 Unit of Parasitology, Department of Biomedicine and Veterinary Public Health, Swedish University of Agricultural Sciences, PO Box 7036, 75007 Uppsala, Sweden

**Keywords:** Demographic history, MaxEnt, Qinghai-Tibet plateau, Chinese grouse, *Tetrastes sewerzowi*, Whole genome sequence

## Abstract

**Background:**

The Quaternary had worldwide consequences in forming the contemporary diversity of many populations, species and communities, which is characterized by marked climatic oscillations between glacial and interglacial periods. The origin and evolution of biodiversity in mountainous areas are highly dependent on historical orogenesis and associated climatic changes. The Chinese grouse *Tetrastes sewerzowi* is a forest-dwelling species endemic to the mountains to the east of the Qinghai–*Tibet* Plateau, which has been listed as Near Threatened with a decreasing trend by the IUCN because of ongoing deforestation and fragmentation of coniferous forests. It is important to place current population status into a broader ecological and evolutionary context to understand their demographic history.

**Results:**

Analyses of the Chinese Grouse genome revealed fluctuations throughout the Pleistocene in effective population size. Populations decreased during early to middle Pleistocene but showed an expansion during late Pleistocene which was then followed by a sharp decline during the last glacial maximum (LGM). Ecological niche modeling indicated that a suitable habitat shift between high altitude regions to low altitude regions was due to a changing climate. This result parallels patterns of population size change in Chinese Grouse estimated from PSMC modelling, which suggested an expansion in population size from the last interglacial period (LIG) and then a peak and a bottleneck occurring at the last glacial maximum (LGM). Furthermore, the present-day distribution of Chinese Grouse is greatly reduced and fragmented. It will likely become even more fragmented in the future since coniferous forest cover is threatened in the region of their distribution and the availability of such habitat restricts their ecological niche.

**Conclusions:**

The Chinese Grouse have experienced substantial population size changes from the beginning to the LIG and reached a peak before the LGM. A sharp decrease and bottleneck occurred during the LGM, when the coniferous forests were subjected to extensive loss. The results inferred from the whole genome sequencing and species distribution models both support historical population fluctuations. The distribution of the Chinese Grouse is strongly dependent on the coniferous forest cover. To protect the fragmented coniferous forests is an essential action to protect the Chinese Grouse.

## Background

The Chinese grouse is a forest-dwelling bird species endemic to the Qinghai–Tibetan Plateau (QTP) [1, 2], which has been listed as *Near Threatened* with a decreasing trend by the International Union for the Conservation of Nature (IUCN) because of ongoing deforestation and fragmentation of coniferous forests [3]. Throughout the range of the species, only the wetter northern slopes of mountain coniferous forests have vegetation that support grouse populations [1]. This results in ongoing significant reductions of population size and area of occupancy when such areas are cut [2]. In addition, there are large-scale deforestation and intensive livestock grazing in QTP area, which exacerbates habitat loss and fragmentation [4]. Furthermore, world climate change possibly threatens the Chinese Grouse not only by increasing habitat loss and fragmentation, but also by influencing range shifts [5, 6].

In mountainous areas, the origin and evolution of biodiversity are highly dependent on the historical orogenesis and past associated climatic changes [7–9]. Complex geological environments and Pleistocene glacial events contributes to complicated refugial patterns, species distributions and the demographic history of populations [9–13]. Four significant glaciations have occurred in the QTP during the Pleistocene, the Xixiabangma (Early Pleistocene), Nyanyaxungla (Middle Pleistocene; 0.78–0.50 million years ago), Guxiang (late Middle Pleistocene) and the last glaciation, including two glacial stages with the last corresponding to the LGM [10, 14, 15]. The coniferous forests declines and expansions coincide with the glaciations. Cold and dry weather conditions happened in the glaciation period, which resulted in decline of coniferous forests. On the contrary, warm and wet weather conditions followed the retreat of the glaciers which were ideal for the spread of coniferous forests. Pollen data and analyses of past animal faunas show that the forest and forest steppe including spruce and fir connected the QTP and the Siberian taiga by the loess plateau in northern China during the Mid Pleistocene [16]. The distribution of existing Palearctic realm taiga birds and mammals, such as Black Woodpecker (*Dryocopus martius*), Spotted Nutcracker (*Nucifraga caryocatactes*), Eurasian Jay (*Garrulus glandarius*), Snowy-browed Nuthatch (*Sitta villosa*), Red Deer (*Cervus elaphus*) support this past connection. A large altitudinal gradient across the region spanning from 500 to 8848 m is created by the uplift of the QTP [17]. The eastern edge of the QTP are now characterized mainly by a warm and wet climate associated with deep valleys and [18].

The Quaternary had worldwide consequences in forming the contemporary diversity of many populations, species and communities, which is characterized by marked climatic oscillations between glacial and interglacial periods that [19–22]. The effect of the Pleistocene climate fluctuations on species distributions and diversity is well known in Europe [23] and North America [24]. The periodic uplift of the QTP before the Pleistocene has contributed to geologically induced complex environments where Pleistocene glaciations formed many relatively small alpine glaciers and refugia [10, 25, 26]. As a consequence, more complex glacial effects may have influenced biotic speciation, distribution and diversity in the QTP compared to Europe and North America [10, 11]. Before the LGM, the warm and wet weather during the “Greatest lake period” (25,000–40,000 years ago) in this region could have contributed to the expansion of the coniferous forests [27–29]. Compared to today, the temperature was 2–4 °C higher and precipitation was 40–100% higher [27]. Furthermore, pollen-based vegetation reconstruction shows that at this time [28, 30] the alpine coniferous forest extended beyond its present western limit about 400–800 km. This expansion of the alpine coniferous forests, the primary habitat for the Giant Panda (*Ailuropoda melanoleuca*) as well as the Chinese Grouse, have resulted in a second population expansion of Giant Pandas [1, 12]. The Chinese Grouse as well as the other typical forests-dwelling birds and mammals should have had similar demographic histories during this period.

The aim of the present study is to infer the past population dynamics of Chinese Grouse by applying Partial Sequential Markov Chain (PSMC) modelling on whole genome sequence data. The PSMC model infer the time of the most recent common ancestor for each independent DNA segment and infer ancestral effective population size (*Ne*) at a given epoch through a hidden Markov framework and identifies historical recombination events across a single diploid genome [31]. Combined with glacial event data, we aim at understanding the effects of past climatic changes on past distribution changes of Chinese Grouse via Species Distribution Modelling (SDM). The combination of SDM and population genomics also provides new insights to understand the impact of past climatic changes on population dynamics. At present, human interference activities had significantly changed climate and habitats in the QTP of southwest China, which have already had, and will continue to cause, untold biodiversity loss [32]. We also performed a GAP analysis, a Geographic Approach to Protection of Biological diversity [33], between present forests and climate driven habitat to inspire future work in the conservation of Chinese Grouse and other forest species in the QTP.

## Results

### De novo assembly of the Chinese grouse genome

The Illumina sequencing had total 184.34 G PE 150 bp reads and the average depth was 184 X. The Pacbio long-read Sequencing had total length of 36.7 GB and the depth was 36 X. The software DBG2OLC produced a reference de novo genome assembly which comprises 1272 scaffolds that had a total length of 1,003,232,344 bp, corresponding to 91% of the total genome length of the expected 1.1 G. Scaffold N50 was 2.16 Mb, and the longest scaffold measured 5.27 Mb. The BUSCO complete assessment score is 93%. For comparison, the well assembled Chicken (*G. gallus*) genome is 1054.6 Mb [34], which is similar with the genome size of Chinese Grouse in the current project.

### Demographic history

**Fig. 1:**
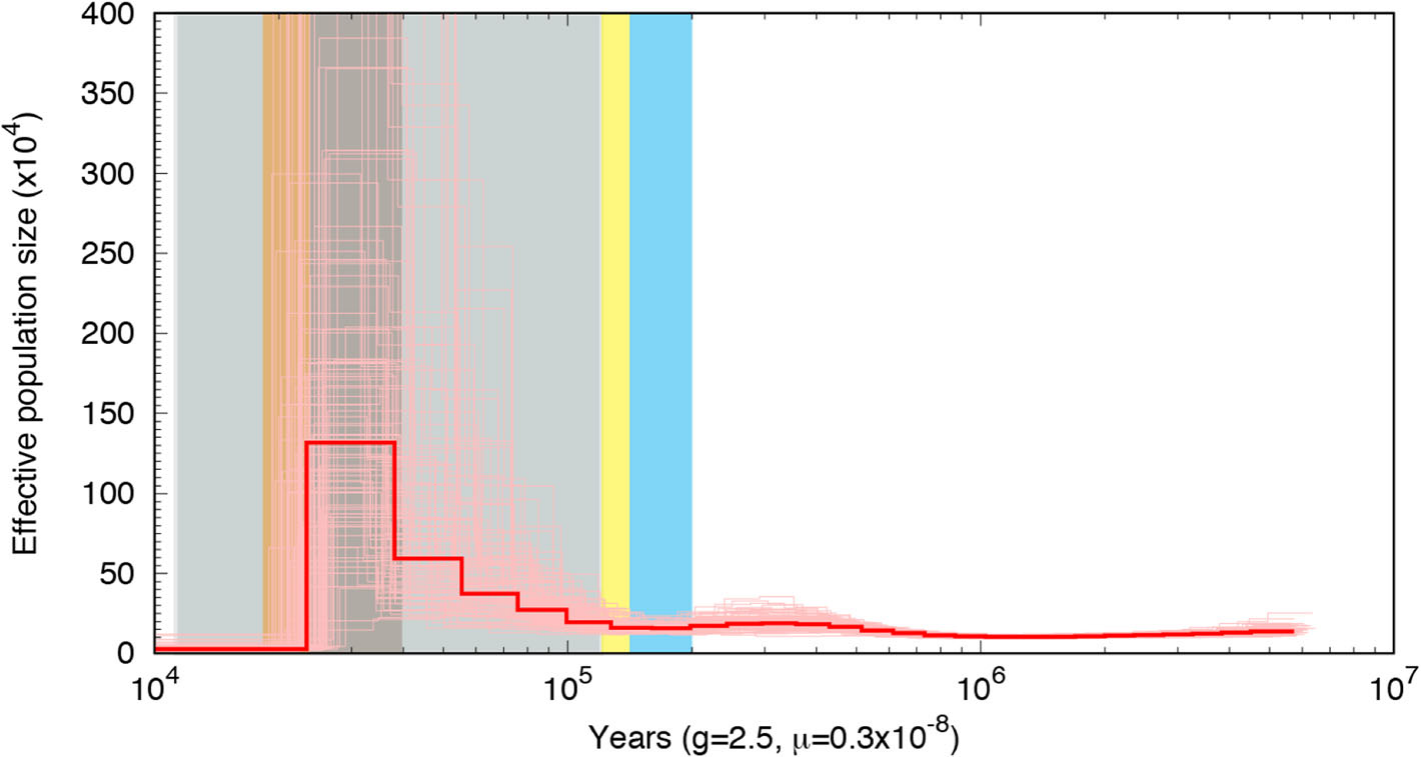
PSMC plot showing the demographic history of Chinese Grouse. The red line represent the estimated effective population size (*N*_*e*_), and 100 thin curves represent the PSMC estinates for 100 sequences randomly resampled from the original sequence.Coloured bars corespond to the climatic periods. Blue: Pennultimate Glacial Period, yellow: The last interglacial (Emain), light grey: last glacial period (LGP), dark grey: the last Greatest Lake Period (GLP), orange: LGM

The simulated demographic history of Chinese Grouse was inferred by plotting the population size fluctuation curve against time with superimposed glacial periods [15]. Chinese Grouse effective population size increased from the time of the last interglacial (around 115 kya ago). Before this time, the effective population size of Chinese Grouse was kept at a high number (20 × 10^4^) from the beginning of the simulations. The results indicated that Chinese Grouse population size reached a peak before the last glaciation Maximum (40–100 kya ago), experienced a significant decrease during the last glaciation Maximum (LGM; ~ 20 kya ago) (Fig. 1), when substantial alpine glaciations (such as Gongga glacial II) would likely have resulted in extensive loss of coniferous forests.

### Ecological niche modeling

**Fig. 2:**
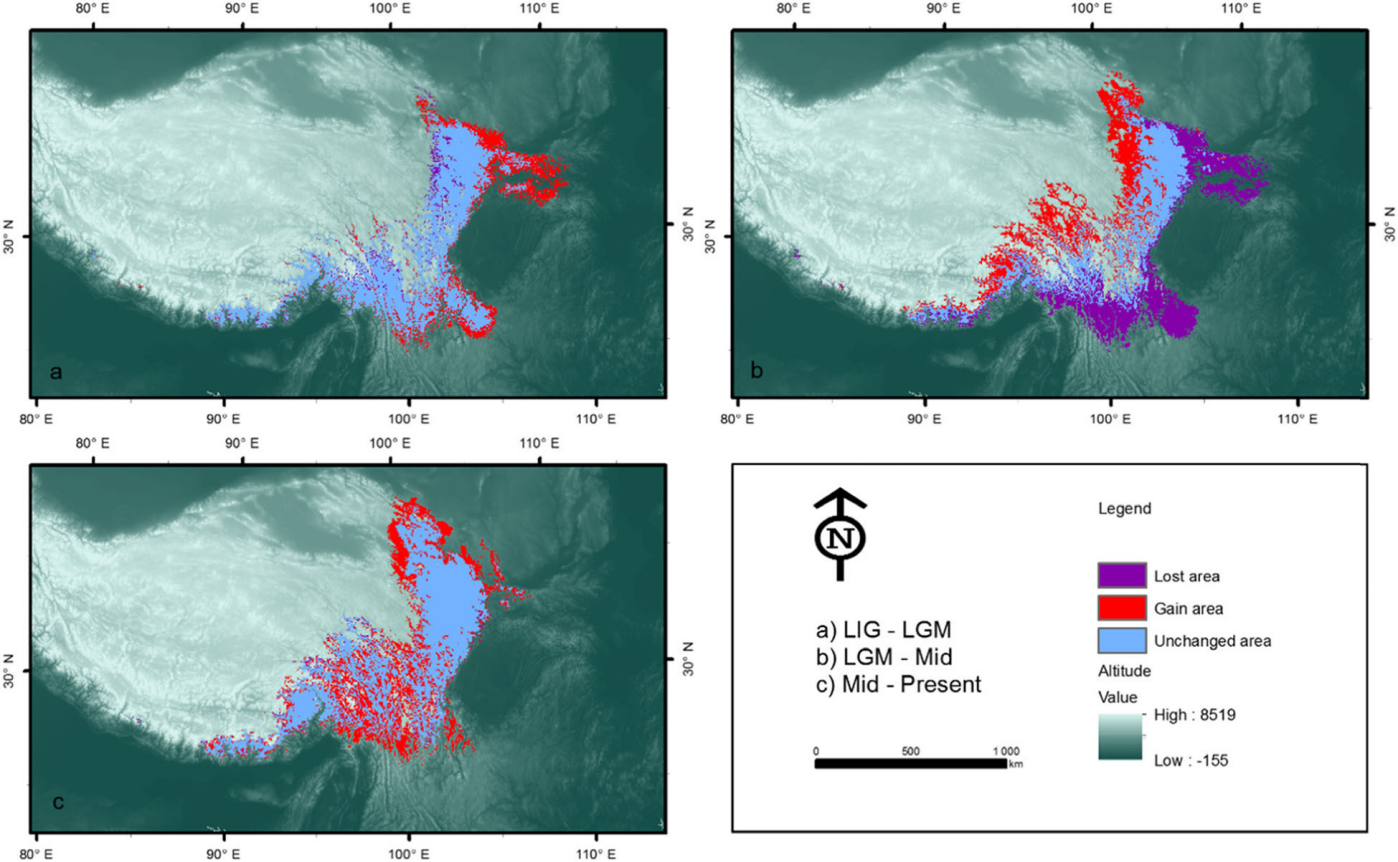
The change in predicted distributions of the Chinese Grouse: **a** from LIG to LGM; **b** from LGM to Mid-Holocene; c predicted from Mid-Holocene to Present-day. Map data was from WorldClim-Global Climae Data, which is free data for ecological modeling and GIS. The map was created by ArcGIS 10.5, the URL link is https://support.esri.com/zh-cn/products/desktop/arcgis-desktop/arcmap/10-5

**Fig. 3:**
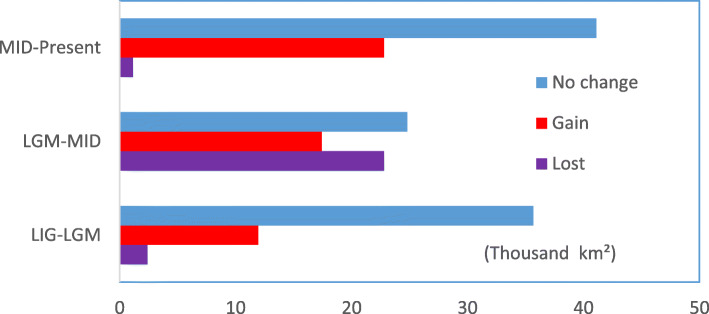
Predicted distribution changed from LIG to LGM, LGM to MID and MID to Present

To assess the role of climate change, we used SDMs and palaeoclimatic data on temperature and precipitation to estimate the suitable habitat of Chinese Grouse during the time periods of 120–140 kya (LIG), 21 kya (LGM), 6 kya (Mid-Holocene), and the present (Fig. 2, Additional file 2). All four SDMs suggest that past climate change may have contributed significantly to the distribution change and population dynamics of Chinese Grouse. The model revealed that suitable distribution during the colder LGM was more widely available than during the warmer LIG and warmer mid Holocene (Fig. 2). Specifically, compared with the LIG period, the area of suitable distribution during the colder LGM period had increased about 11. 9375 thousand km^2^ and decreased 2.3875 thousand km^2^ (Fig. 2a, Fig. 3). The expansion of suitable distribution was moved eastwards which was a low elevation area. On the contrary, the area of suitable distribution during LGM period was less compared with the Mid Holocene period (Fig. 2b). The suitable distribution during Mid Holocene moved to westwards which was at high elevation in the Tibetan plateau and the former eastern area was lost (Fig. 2b).

**Fig. 4:**
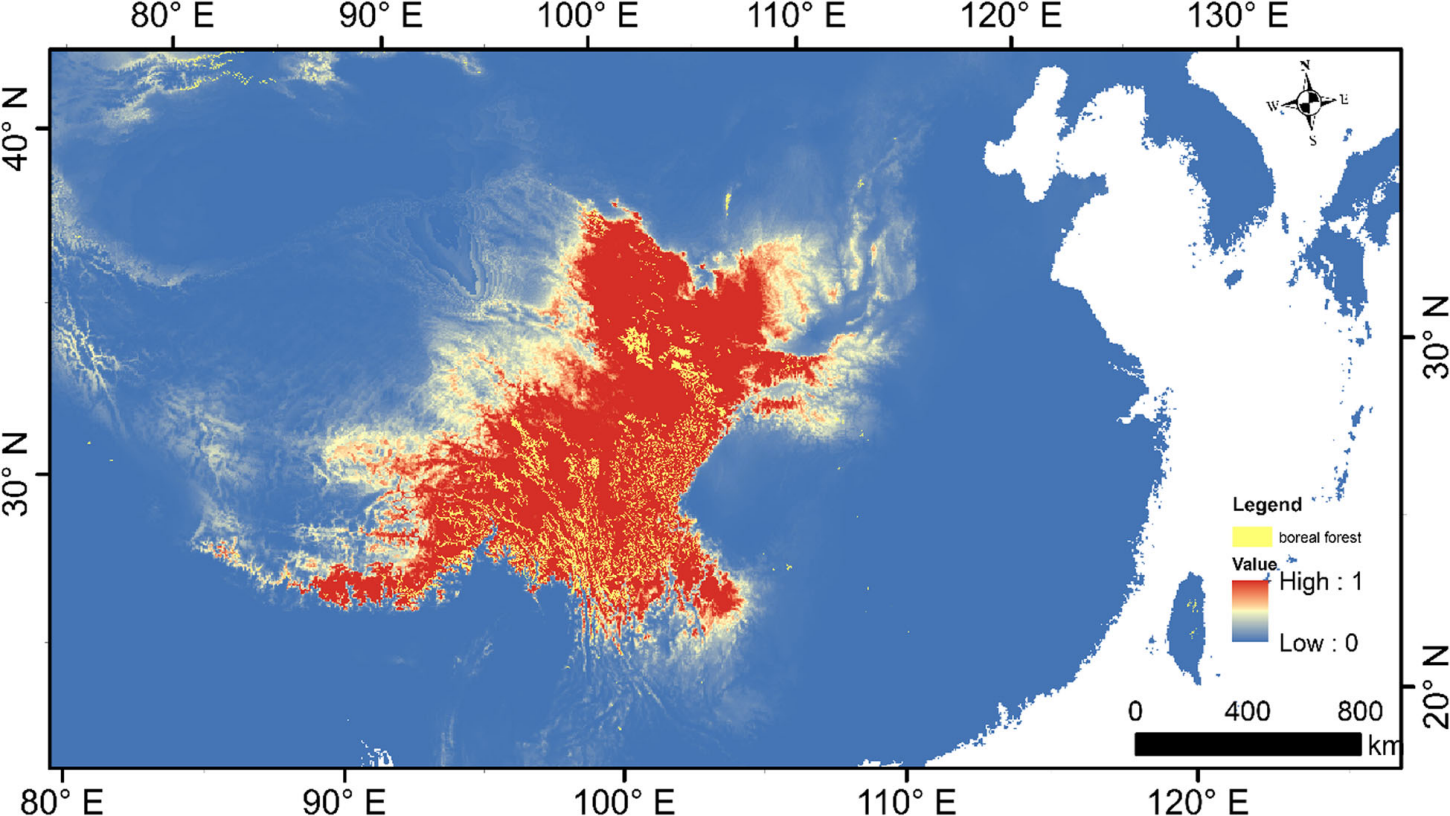
The GAP analysis of predicted distribution of the Chinese Grouse in the present-day (colour bar) and boreal forest cover (the yeallow area). Colours represents the suitability, from low (blue) to high (red). Map data was from WorldClim-Global Climae Data, which is free data for ecological modeling and GIS. The map was created by ArcGIS 10.5, which URL link is https://support.esri.com/zh-cn/products/desktop/arcgis-desktop/arcmap/10-5

The population size change in Chinese Grouse estimated from the PSMC model suggested an expansion in population size during the LIG and a bottleneck occurring at the LGM. Strikingly, the suitable distribution of Chinese Grouse expanded toward warmer eastern areas during the LGM and moved to higher and western areas during Mid Holocene. Compared with the Mid Holocene period, the predicted present-day distributions is increased and concentrated predominantly in the model only conducted in climate change scenario (Fig. 2c, Fig. 3). But the present-day distribution of Chinese Grouse was greatly reduced and become highly fragmented after the GAP analysis was performed between the predicted present-day distributions and coniferous forest cover (Fig. 4).

## Discussion

The demographic analyses showed the grouse population reached a peak ~ 40,000 years ago followed by sharp decrease with a bottleneck ~ 20,000 years ago. The population expansion can be explained by the warmer weather during the Greatest Lake Period (30,000–40,000 years ago) as the conifer forests, the primary habitat for Chinese Grouse [1], reached their greatest extent at this time [10, 12]. The Palaeo-distribution of coniferous forests changes in the QTP and adjacent mountains inferred by pollen cores [35] support the demographic changes inferred of Chinese Grouse [35, 36]. The demographic results and palaeo coniferous forests data show that coniferous forests played a pivotal role in Chinese Grouse demographic history by providing permanent refugias in the eastern QTP during the Quaternary. The QTP with its special intricate geographical environment had a significant influence on biodiversity and changes in the geographic distribution of animals in east of Asia during the Pleistocene and may play a key role on potential responses and feedbacks to global change in the future [11, 37].

Our demographic analyses highlighted that a high Ne of Chinese grouse persisted from the Early Pleistocene (2.43 million years ago-0.73 million years ago) to the Mid Pleistocene (0.73 million years ago - 0.10 million years ago) and that a population expansion occurred after the retreat of the Penultimate Glaciation (0.30–0.13 million years ago). Three significant glaciations have occurred in QTP during this time, the Xixiabangma glaciation in Early Pleistocene, Nyanyaxungla glaciation in Middle Pleistocene and Guxiang glaciation in late Middle Pleistocene [14, 15]. Like previous work, our results suggest that the QTP had several Pleistocene refugia which have affected Ne in this grouse inhabiting coniferous forests [10]. During the Pleistocene, many avian species living in coniferous forest in the boreal taiga and the QTP have diverged and are classified as superspecies or different subspecies, as a result of allopatric divergence and local adaptation [8, 23, 38, 39]. A narrow high altitude boreal forests were preserved in the southeast edge of the QTP by the uplift which comprise one of the key high-altitude biodiversity hotspots in the world [40]. This region harbors one of the world’s richest fauna and floras, because it harbored large and complicated refugial areas in different mountain regions, lowland, rivers and basins [10, 41, 42].

When substantial alpine glaciations (for example, Gongga glacial II) likely resulted in extensive loss of coniferous forests, a population bottleneck occurred during the last glacial maximum (~ 20,000 years ago), [15]. Population bottlenecks in the same region coinciding with the LGM have also been identified in other animals such as the giant panda [12] and snub-nosed monkeys (*Rhinopithecus*) [13], suggesting that the Last Glaciation and palaeo coniferous forests changes had strong impacts on the population sizes of arboreal species. The sharp decline in Ne for Chinese Grouse, as in the Giant panda, snub-nosed monkeys, is consistent with the extreme cooling of the climate during the LGM.

Many previous studies have suggested that the LIG was a favorable period for other avian species, such as black grouse (*Tetrao tetrix*) because of increased seasonality in temperatures [43, 44]. During this period, black grouse and Greenland rock ptarmigan (*Lagopus muta*) populations expanded to a high N_e_ and reached their peaks [43, 45]. It is suggested that the severe reduction in avian population sizes approximately coincide with the beginning of the last glacial period (LGP, 110–12 kya) or occurring during this period [44]. In contrast to previous work, the demographical results of Chinese Grouse show no fluctuations associated with historical climatic changes during Early Pleistocene and Middle Pleistocene [44]. Instead a peak followed by a sharp decrease and a bottleneck occurred during this period. This pattern observed in Chinese Grouse may be related to the special geographical position, climate change and the mass accumulation rate (MAR) of Chinese loess, an index indicating cold and dry or warm and wet climatic periods in China [46, 47].

The SDM results showed that climate change had an influence on the amount and distribution shifts of suitable habitat of Chinese grouse. The model also revealed that suitable habitat during the LGM was more widely available than both during the warmer LIG and mid Holocene. Climate events during the LGM period resulted in an expansion of suitable habitat to low altitude regions in eastward areas compared with the LIG period and a shrink from this low altitude region to a more westward distribution during the mid-Holocene period. The result parallels patterns of population size change in Chinese Grouse estimated from the PSMC model, which suggested an expansion in population size from LIG period and then a peak and a bottleneck occurring at the LGM. Significantly, the suitable habitat of Chinese Grouse expanded toward lower altitudes in eastern areas during the LGM. In contrast, the population moved into high altitudes in the western areas during the mid-Holocene. It is of significance that the geographic distribution of Chinese Grouse populations shifted in an east-west direction from LIG to the LGM and the LGM to mid-Holocene. The reason is that the shift of coniferous forests was significantly affected by seasonal extremes in temperature. In addition, the formation of the loess plateau stopped the forest to spread to the north.

Alarmingly, our results suggest that although suitable areas for the Chinese Grouse in the present-day are larger than during the mid-Holocene, the LGM and the LIG, the Ne has been kept at a low size after the bottleneck during the LGM. Dense coniferous forests are of critical importance for the Chinese Grouse, which are affected by climate change and recent human actions [1]. Our GAP analysis between the present-day distribution of Chinese Grouse and extent of present day coniferous forest shows that the Chinese Grouse populations were severely affected by loss and fragmentation of suitable forests. Forest fragmentation has profound and lasting influences not only on the Earth’s biological diversity, but on ecosystem function and numerous ecosystem services [48, 49]. The forest in the QTP started to become protected from 1998 when a disastrous flooding happened. Because of the increase in density of local people with the high demands for farmland, timber, firewood and roads, deforestation of Chinese grouse’s mountain habitats continued [1, 6]. Previous work on Chinese Grouse, blood pheasant (*Ithaginis cruentus*) and Sichuan jay (*Perisoreus internigrans*) have found that their distributions would be decreased and fragmented severely in the future [5, 6, 50]. The Chinese Grouse distributions under climate change scenarios is similar to other montane species [51], which would shift northward and upward under realistic climate change scenarios [6]. This long term fragmentation of coniferous forests may have effect on gene flow, speciation and divergence of these endemic isolated populations.

## Conclusion

The Chinese Grouse have experienced substantial population size changes from the beginning to the LIG and reached a peak before the LGM. A sharp decrease and bottleneck happened during the LGM, when the conifer forests were subjected to extensive loss. The results inferred from the whole genome sequencing and species distribution models both support a history of population size fluctuations. The distribution of the Chinese Grouse were strongly dependent on the coniferous forest cover. To protect the fragmented such forests is an essential action to protect the Chinese Grouse.

## Methods

### Sampling information

A blood sample was collected from one individual from the Lianhuashan Nature Reserve, Gansu province, China. The sample collection was conform to the National Wildlife Conservation Law in China and with permission from the Forestry Department of China. Our sample collection procedures also followed the regulations of the animal experimental and medical ethics committee of the Institute of Zoology, Chinese Academy of Sciences. The bird was released after the blood sample collection.

### De novo genome sequencing and data production

We used a Gentra Puregene Blood Kit (Qiagen) to extract the genomic DNA from a whole blood sample according to the manufacturer’s instructions. Then we assessed the quality of DNA by electrophoresis on 1% agarose gel and the quantity of DNA by a BioDrop mLITE pectrophotometer (a total of 15 mg of DNA was quantified using the spectrophotometer). The sequencing included two platforms: Illumina (San Diego, CA) Hiseq4000 and Pacific Biosciences (Menlo Park, CA) PacBio RSII. We used Illumina DNA Sample Prep Kit to construct three paired-end libraries with insert size of 350 bp and SMRT bell library preparation protocol (10 SMRT cells were sequenced) to generated by Pacific Biosciences long reads (> 10 kb).

### Assembly

We removed contaminated reads with Illumina paired-end adaptors using TRIMMOMATIC 0.36 [52] with the options ILLUMINACLIP: TRUSEQ3-PE.FA: 2: 30: 10 MINLEN: 150. Then bad reads were eliminated based on thresh hold of: base quality lower than Phred score of 20; N content above 10%; base quality below 20 proportion above 50%. This allowed us to only keep high-quality paired end reads for the assembly steps.

We estimated the best k-mer length for genome de novo assembly by KmerGenie. We used a hybrid assembly strategy following the protocol of DBG2OLC toolset [53]. The parameter: LD 0 k 57 g 25 NodeCovTh 2 EdgeCovTh 1 GS 4000000000 were employed during SparseAssembler step to process the illumina paired-end reads. K 57 was chosed according to k-mer estimation of KmerGenie. GS was set to about 4 folds of conventional avian genome size 1G for memory usage management. The preliminary contigs file was passed to DBG2OLC to make layout with Pacbio long SMRT reads. LD 1 k 49 AdaptiveTh 0.005 KmerCovTh 5 MinOverlap 30 RemoveChimera 1 were used to generate raw backbone. Finally, blasr and consensus modules were implemented to produce the genome assembly. The final assembled genome was polished by pilon-1.22 [54] to detect possible misassemblies. The final assembly was used for downstream analysis.

### Demographic history inference using PSMC

To reconstruct the demographic history of Chinese Grouse, we used the Pairwise Sequentially Markovian Coalescent (PSMC 0.6.5) [31] model to infer the effective population sizes (Ne) of Chinese Grouse across genome sequences with SNP sites. We used the consensus autosomal sequence (in *fastq* format) for Chinese Grouse as the input for the PSMC modelling. To run the PSMC method, we used two parameters: the generation time (2.5 years) and the mutation rate per generation (0.3 × 10^− 8^ per nucleotide per year) of Chinese Grouse. The mutation rate (per nucleotide per year, μ) for Chinese Grouse was selected from studies of willow grouse (0.299 × 10^− 8^) and rock ptarmigan (0.310 × 10^− 8^) [55]. PSMC was run for 100 iterations using an initial h = q ratio of 5 and the default time patterning. Bootstrapping was performed as in Li and Durbin (2011) [31] by resampling 500,000 bp chunks of the genome with replacement to perform 100 bootstrap replicates.

### SDM model

We used the maximum-entropy approach (MaxEnt, ver. 3.3.3 k) [56] to model Chinese Grouse habitat. A set of layers, or environmental variables, as well as a set of georeferenced occurrence data were used to produce a model of the ancient and present range of Chinese Grouse. We projected the present-day and three other periods Species Distribution Models (SDMs) based on the climate data during the current, mid-Holocene (6000 years BP), last glacial maximum (LGM, 21,000 years BP), and last interglacial (LIG, 120,000–140,000 years BP). Our models performed well (AUC value is 0.991) for the Chinese Grouse (Additional file 1). We obtained all the 19 bio-climatic variables from the WorldClim 1.4 database at a spatial resolution of 2.5 arc-minutes [57]. The current climatic data layers were based on spatially interpolated values of temperature and precipitation from 1960 to 1990, whereas the variables were generated from the climate reconstructions based on CCSM4 for both the period mid-Holocene and LGM [57], and the variables for LIG were downscaled from simulations with CCSM4 [58]. We used the same data set of grouse location (observation) records as from a reference by Lu et al. [5, 6]. The 41 location records we used to construct the SDMs were set in a spatial distance threshold of 0.083 decimal degrees (i.e. 5′, about 8 km) which can reduced spatial autocorrelation [59]. Further parameter setting refer to Lu et al. [6]. Furthermore, a 10% training presence logistic threshold was used to define the minimum probability of suitable habitat. A GAP [33] analysis between current distribution and coniferous forests were performed to assess conservation status. All data preparation and raster calculations were performed in ArcGIS 10.5.

## Supplementary information

**Additional file 1.** ROC Plot for classification accuracy of this model.

**Additional file 2.** Predicted distribution of Chinese Grouse in different periods: a, LIG; b, LGM; c, Mid-Holocene’ d, Present day. The grey area represent the distribution in different time and the color bar represent altitude.

## Data Availability

Sequencing data for the Chinese Grouse have been deposited in Short Read Archive under project number PRJNA588719. WorldClim – Global Climate Data is free climate data for ecological modeling and GIS at https://worldclim.org/data/v1.4/worldclim14.html and https://worldclim.org/data/v1.4/paleo1.4.html. These data can be used for mapping and spatial modeling. The data set of grouse location (observation) records is from a paper by Lu et al. 2012. The link is https://www.sciencedirect.com/science/article/pii/S0006320712001863.

## Abbreviations

QTP: Qinghai–Tibetan Plateau
SDM: Species Distribution Modelling
PSMC: Pairwise Sequentially Markovian Coalescent
MID: Mid-Holocene
LGM: Last glacial maximum
LIG: Last interglacial
