# Supplementary figures and images for: Genomic analysis of demographic history and ecological niche modeling in the endangered Chinese Grouse Tetrastes sewerzowi
Source: BMC Genomics. 2020 Aug 27;21:581. doi: 10.1186/s12864-020-06957-5 (PMC7450560; doi:10.1186/s12864-020-06957-5)

**Additional file 1:** ROC Plot for classification accuracy of this model.


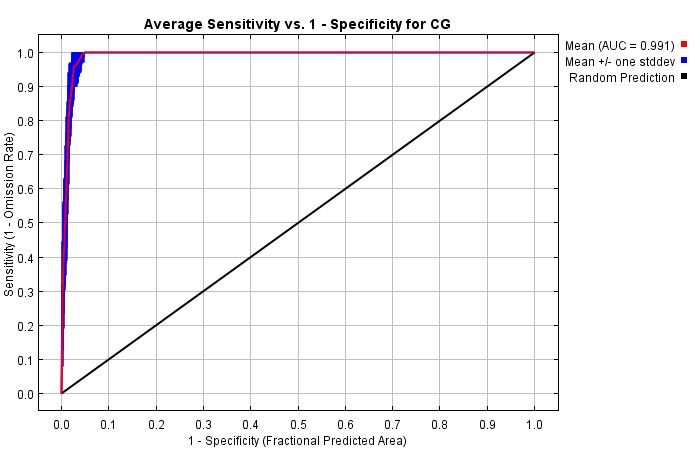

Supplement: Supplementary file 1 — Additional file 1. ROC Plot for classification accuracy of this model. [file 12864_2020_6957_MOESM1_ESM.docx]
