# Supplementary material for: Genomic analysis of demographic history and ecological niche modeling in the endangered Chinese Grouse Tetrastes sewerzowi
Source: BMC Genomics. 2020 Aug 27;21:581. doi: 10.1186/s12864-020-06957-5 (PMC7450560; doi:10.1186/s12864-020-06957-5)

**Additional file 2:** Predicted distribution of Chinese Grouse in different periods: a, LIG; b, LGM; c, Mid-Holocene’ d, Present day. The grey area represent the distribution in different time and the color bar represent altitude. Map data was from WorldClim-Global Climae Data, which is free data for ecological modeling and GIS.


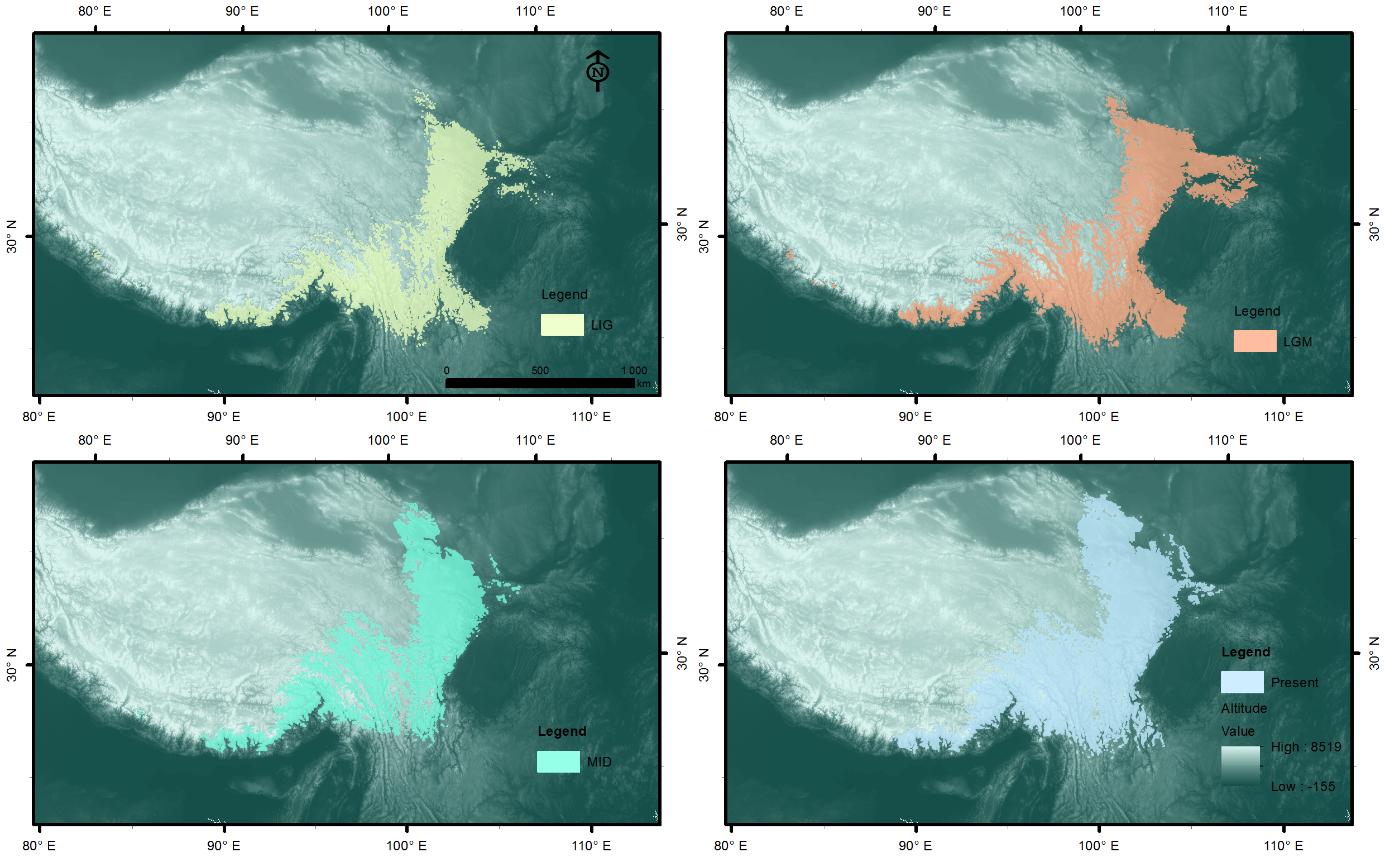

Supplement: Supplementary file 2 — Additional file 2. Predicted distribution of Chinese Grouse in different periods: a, LIG; b, LGM; c, Mid-Holocene’ d, Present day. The grey area represent the distribution in different time and the color bar represent altitude. [file 12864_2020_6957_MOESM2_ESM.docx]
